# What’s new in the 2025 GOLD report

**DOI:** 10.36416/1806-3756/e20240412

**Published:** 2025-03-18

**Authors:** David M G Halpin, Dave Singh

**Affiliations:** 1. University of Exeter Medical School, Department of Health and Community Sciences, Exeter, UK.; 2. Wythenshawe Hospital, University of Manchester, Manchester University NHS Foundation Trust, Manchester, UK.; 3. Wythenshawe Hospital, Medicines Evaluation Unit, Manchester University NHS Foundation Trust, Manchester, UK.

**Figure 1:**
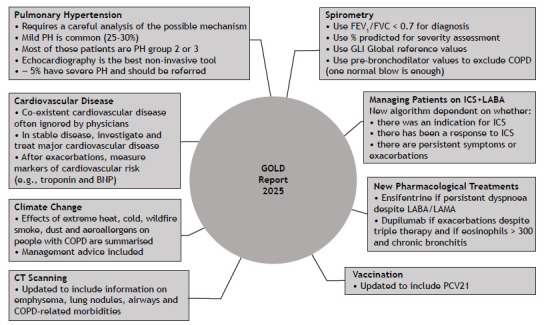
Key updates in the 2025 GOLD report. PH: pulmonary hypertension; GLI: Global Lung Function Initiative; BNP: brain natriuretic peptide; ICS: inhaled corticosteroid(s); LABA: long-acting β2 agonist; LAMA: long-acting muscarinic antagonist; and PCV21: 21-valent pneumococcal conjugate vaccine.

The GOLD reports serve to enable health care professionals to better manage COPD. The GOLD science committee updates the report every year by incorporating the latest evidence relevant to clinical practice, aiming to be as practical and easy to follow as possible. The 2025 GOLD report contains important changes (Figure 1), notably regarding diagnosis and pharmacological management, as well as a new section on climate change and COPD.^1^

The diagnosis of COPD requires initial clinical assessment of respiratory symptoms and exposure to risk factors. Diagnostic confirmation is obtained using spirometry to demonstrate the presence of airflow obstruction, which is defined as an FEV_1_/FVC ratio of < 0.7. The 2025 GOLD report considers the merits of using pre- or post-bronchodilator measurements for this purpose. Large cohort studies have demonstrated that although pre-bronchodilator spirometry and post-bronchodilator spirometry give the same diagnostic results in the majority of individuals, post-bronchodilator values can result in up to 36% fewer diagnoses due to a “flow” response characterised by an increase in FEV_1_ that pushes FEV_1_/FVC > 0.7.^2^ However, administration of a bronchodilator can reduce gas trapping (“volume” response). This improves FVC, thereby reducing the FEV_1_/FVC ratio; there are a small number of “volume” responders who move from > 0.7 to < 0.7 after bronchodilator administration.^2^ The 2025 GOLD report recommends using pre-bronchodilator spirometry > 0.7 to rule out COPD, unless a volume responder is suspected on the basis of low FEV_1_ or a high symptom burden. This recommendation can avoid unnecessary post-bronchodilator spirometry being performed. If pre-bronchodilator spirometry is < 0.7, then post-bronchodilator measurements are needed for diagnostic confirmation. Flow responders who move to > 0.7 after bronchodilator administration have a high prevalence of developing COPD over time and need careful prospective monitoring.^3^ There has been considerable debate concerning the use of the fixed ratio (0.7) versus lower limit of normal (LLN) values (which classify the bottom 5% of the healthy population as abnormal) for diagnostic purposes. The 2025 GOLD report includes some discussion on this issue. The LLN depends on the reference equation used, which are mostly based on pre-bronchodilator values that will over-estimate the number of cases.^2^ ^,^ ^4^ On the basis of simplicity for a worldwide diagnostic test and the fact that there is no absolute right or wrong, GOLD continues to recommend the use of the fixed ratio over the LLN. 

Clinical trials in COPD patients with a history of exacerbations in the previous year have consistently demonstrated superiority of triple therapy over the combination of an inhaled corticosteroid (ICS) and a long-acting β_2_ agonist (LABA) for exacerbation prevention, lung function and quality of life.^5^ ^,^ ^6^ Exacerbations have important detrimental effects on other outcomes, including prolonged impaired quality of life, greater lung function loss and increased mortality. Given the clinical importance of exacerbation prevention, GOLD recommends triple therapy over the ICS-LABA combination if treatment with ICS is indicated. For patients who have been historically treated with the ICS-LABA combination, there is an opportunity to optimise treatment. The 2025 GOLD report includes a new algorithm to help decide the next step, which may include escalation to triple therapy for patients who currently have exacerbations and have blood eosinophil counts > 100 cells/µL (a marker of corticosteroid-sensitive inflammation). For patients who are not currently exacerbating, it is crucial to understand whether there was no prior history of exacerbations, and therefore inappropriate use of ICS, or if previous exacerbations responded to ICS treatment, because this changes the next step. 

The 2025 GOLD report includes recommendations on two novel classes of medications to treat COPD: a dual phosphodiesterase 3 (PDE3)/phosphodiesterase 4 (PDE4) inhibitor and the first biologic therapy to be approved for COPD. The inhaled PDE3/PDE4 inhibitor ensifentrine has both anti-inflammatory activity and bronchodilator effects. It significantly improved lung function and dyspnoea but had inconsistent effects on quality of life in parallel phase III studies^7^; however, the studies did not assess the impact of ensifentrine on top of LABA plus a long-acting muscarinic antagonist (LAMA) or LABA+LAMA+ICS, making it difficult to assess the relevance of its effects on exacerbations when positioning it in the treatment algorithm. The 2025 GOLD report recommends that ensifentrine be added to dual bronchodilator therapy if the patient continues to experience dyspnoea. 

Dupilumab is a fully human monoclonal antibody that blocks the shared IL4 and IL13 receptor. It reduced exacerbation rate and improved lung function and health status in two large randomised trials.^8^ ^,^ ^9^ The patients in those studies all had chronic bronchitis; a history of two or more moderate exacerbations or one or more severe exacerbations in the last year despite treatment with LABA+LAMA+ICS; and blood eosinophil counts ≥ 300 cells/µL. Reflecting the trial entry criteria, the 2025 GOLD report recommends that dupilumab be added to triple therapy if patients continue to have exacerbations and have a blood eosinophil count ≥ 300 cells/µL and symptoms of chronic bronchitis. 

It is well known that the prevalence of cardiovascular disease is high in COPD patients. Cardiovascular disease often goes unnoticed and untreated in patients with COPD.^10^ Clinicians are perhaps less aware that the risk of cardiovascular events, including myocardial infarction and stroke, increases during and after an exacerbation.^10^ ^,^ ^11^ Although the mechanisms remain to be fully elucidated, systemic inflammation and hypoxia are likely to play key roles in causing cardiovascular stress. A post-hoc analysis has recently demonstrated that triple therapy reduces cardiovascular events in comparison with LAMA/LABA, presumably through exacerbation prevention.^12^ The 2025 GOLD report includes a new section on cardiovascular risk, with the aim of raising awareness and encouraging proactive investigation and therapeutic intervention. It also includes a more detailed section on pulmonary hypertension and its investigation and management in patients with COPD, as well as updated sections on vaccination and the role of CT scanning in assessing emphysema, lung nodules, airways and COPD-related comorbidities. 

The 2025 GOLD report includes a new section on climate change and the impact of the more frequent and extreme weather events it has caused on people with COPD. Extreme heat and cold are both associated with an increased risk of death in people with COPD,^13^ ^-^ ^15^ with the risk being greater with cold.^16^ ^,^ ^17^ High outdoor temperatures are also associated with an increased risk of hospitalisation for COPD,^15^ ^,^ ^18^ ^,^ ^19^ as well as with increased dyspnoea and use of short-acting β_2_ agonists,^20^ ^,^ ^21^ whilst lower outdoor temperatures are associated with an increased risk of exacerbations, increased cough and sputum, increased use of short-acting β_2_ agonists and a fall in FEV_1_.^20^ ^,^ ^22^ ^-^ ^25^ Weather also has a significant impact on air quality, and several studies have examined the interactive effects of pollution and temperature in people with COPD. There appears to be a greater effect of pollutants on COPD hospital admissions and emergency visits at low temperatures or during winter.^26^ ^-^ ^28^

GOLD recommends that patients keep adequately hydrated, keep out of the heat and try to keep living spaces at temperatures of < 32°C and sleeping spaces at temperatures of < 24°C during heatwaves, as well as keeping bedroom temperatures above 18°C during cold weather, as recommended by the WHO. Prior identification and management of cardiovascular comorbidities are also important to reduce adverse outcomes. The 2025 GOLD report also points out that the selection of inhalers and the correct disposal of inhalers by patients can have important implications for global warming and climate changes, and these should be considered when prescribing therapy. 

The GOLD reports provide recommendations on the diagnosis and assessment of patients with COPD, as well as comprehensive recommendations on the management of stable disease, exacerbations and comorbidities. The updates and additions in the 2025 report ensure that these reflect the current evidence base and include newly available treatment options. 
